# Systemic Delivery of Oncolytic Adenovirus to Tumors Using Tumor-Infiltrating Lymphocytes as Carriers

**DOI:** 10.3390/cells10050978

**Published:** 2021-04-22

**Authors:** Joao Santos, Camilla Heiniö, Dafne Quixabeira, Sadia Zafar, James Clubb, Santeri Pakola, Victor Cervera-Carrascon, Riikka Havunen, Anna Kanerva, Akseli Hemminki

**Affiliations:** 1 Cancer Gene Therapy Group, Faculty of Medicine, Translational Immunology Research Program, University of Helsinki, 00290 Helsinki, Finland; joao.santos@helsinki.fi (J.S.); camilla.heinio@helsinki.fi (C.H.); dafne.quixabeira@helsinki.fi (D.Q.); sadia.zafar@helsinki.fi (S.Z.); james.clubb@helsinki.fi (J.C.); santeri.pakola@helsinki.fi (S.P.); victor.cerveracarrascon@helsinki.fi (V.C.-C.); riikka.havunen@helsinki.fi (R.H.); anna.kanerva@helsinki.fi (A.K.); 2 TILT Biotherapeutics Ltd., 00290 Helsinki, Finland; 3 Department of Obstetrics and Gynecology, Helsinki University Hospital, University of Helsinki, 00290 Helsinki, Finland; 4 Helsinki University Hospital Comprehensive Cancer Center, 00290 Helsinki, Finland

**Keywords:** tumor-infiltrating lymphocytes, oncolytic virus, systemic delivery, cell carrier

## Abstract

Immunotherapy with tumor-infiltrating lymphocytes (TIL) or oncolytic adenoviruses, have shown promising results in cancer treatment, when used as separate therapies. When used in combination, the antitumor effect is synergistically potentiated due oncolytic adenovirus infection and its immune stimulating effects on T cells. Indeed, studies in hamsters have shown a 100% complete response rate when animals were treated with oncolytic adenovirus coding for TNFa and IL-2 (Ad5/3-E2F-D24-hTNFa-IRES-hIL2; TILT-123) and TIL therapy. In humans, one caveat with oncolytic virus therapy is that intratumoral injection has been traditionally preferred over systemic administration, for achieving sufficient virus concentrations in tumors, especially when neutralizing antibodies emerge. We have previously shown that 5/3 chimeric oncolytic adenovirus can bind to human lymphocytes for avoidance of neutralization. In this study, we hypothesized that incubation of oncolytic adenovirus (TILT-123) with TILs prior to systemic injection would allow delivery of virus to tumors. This approach would deliver both components in one self-amplifying product. TILs would help deliver TILT-123, whose replication will recruit more TILs and increase their cytotoxicity. In vitro, TILT-123 was seen binding efficiently to lymphocytes, supporting the idea of dual administration. We show in vivo in different models that virus could be delivered to tumors with TILs as carriers.

## 1. Introduction

Since its first steps in the form of Coley’s famous toxin experiments in the early 1890s, the field of immunotherapy has taken huge leaps forward. Checkpoint antibodies have already transformed the field of oncology, while other forms of immunotherapy, like oncolytic viruses and tumor infiltrating T cell therapy (TIL therapy), are emerging rapidly. Rosenberg and colleagues were front runners in the development of TIL therapy [1]. They developed an adoptive T cell therapy program based on TIL therapy, where they demonstrated durable responses in metastatic melanoma. However, questions remain if TIL efficacy and safety can be improved, especially since the pre- and post-conditioning needed for efficacy are toxic [2]. In a meta-analysis TIL therapy in combination with low or high-dose of recombinant interleukin-2 (IL-2) for treatment of advanced cutaneous melanoma, Dafni et al. reported a pooled overall response rate (ORR) of 41% and an overall complete response rate (CR) of 12%. Separately, the ORR in the high-dose IL-2 group was 43%, while for the low-dose IL-2 35% was reported [3]. In non-melanoma cancer types, response rates appear lower, therefore requiring additional work to unlock the full potential of TIL therapy.

It has been noted that one pitfall of T-cell therapies is tumor structures, such as the extracellular matrix that inhibits T cells from penetrating the tumor. T cells can also be inactivated by an unfavorable tumor microenvironment, containing immunosuppressive cytokines or other suppressive stromal cells [4]. Addressing these issues might enhance response rates. Oncolytic adenoviruses could alleviate this issue by creating a favorable tumor microenvironment through the release of danger and pathogen associated molecules combined with activating cytokines and chemokine expression during virus infection [5,6]. Additionally, viruses aid in tumor destruction by direct lysis of the cancer cells [5,7].

The positive effects of combinatorial treatments with therapeutic viruses and T cells have also been noted in a clinical setting. Indeed, Dummer and colleagues have shown that a non-replicating IL-2-encoding adenovirus could improve clinical responses of melanoma patients receiving TIL therapy [8]. More recently, a phase Ib study of the combination of talimogene laherparepvec (oncolytic Herpes virus) with T-cell stimulating anti-PD-1 antibody pembrolizumab has been reported [9]. The therapy was generally well tolerated in 21 patients, with only fatigue, fevers, and chills as the most common adverse events. No dose-limiting toxicities occurred. Confirmed objective response rate was 62%, with a CR rate of 33% per immune-related response criteria. Patients who responded to combination therapy had increased CD8^+^ T cells, elevated PD-L1 protein expression, as well as IFN-γ gene expression on several cell subsets in tumors after talimogene laherparepvec treatment. Response to combination therapy did not appear to be associated with baseline CD8^+^ T cell infiltration or baseline IFN-γ signature [9]. These findings suggest that virotherapy may improve the efficacy of T-cell-based therapies by altering the tumor microenvironment [9,10].

Such promising clinical results employ, however, intratumoral (i.t.) administration of virus, which may be challenging for routine application in some clinical centers. Alternatively, while technically easy, intravenous (i.v.) injection of oncolytic viruses faces its own challenges. Viruses can be opsonized by neutralizing antibodies and removed by immune cells upon infusion [11]. Additionally, macrophages of the liver and spleen can remove viruses from blood [12]. Moreover, much of the virus dose will end up in non-target organs following systemic administration. All these factors limit tumor transduction, which is why oncolytic viruses are typically administered i.t. We previously showed that 5/3 chimeric oncolytic adenoviruses can bind the grooves of white blood cells for potential avoidance of above-mentioned clearance mechanisms [13]. Thus, here we aimed to determine if T-cells could be used to transport clinically relevant adenoviruses to tumors, and if combinatorial injection could result in synergy manifesting in better antitumor efficacy than standard administration of oncolytic virus i.t. and T-cells i.v.

Our laboratory has previously generated a cytokine-encoding oncolytic adenovirus (Ad5/3-E2F-d24-hTNFa-IRES-hIL2 or TILT-123) [14], which was used in this study. The virus resulted from extensive studies determining which cytokines administered to the tumor microenvironment could alter it favorably with regard to the tumor immune component [15]. This study led to tumor necrosis factor alpha (TNFa) and IL-2 to be chosen and inserted into an oncolytic adenovirus, with mutations for cancer cell selectivity (E2F tumor-specific promoter, D24 deletion in the E1A gene) and enhanced cancer cell entry by knob protein exchange (Ad5 knob was exchanged to serotype 3 knob). TILT-123 was demonstrated active in transgene expression (TNFa and IL-2) and oncolytic potential in human and hamster tumor types. As it was shown that TILT-123 works synergistically with T cell therapy, curing 100% of the animals [16], our next hypothesis was that we could deliver both components as one product in one injection. Consequently, this should lead to: (1) higher virus load in the tumor, (2) more virus-mediated lysis of cancer cells, which produce TIL enhancing cytokines, and, eventually, (3) better therapy results with more activated and mature TILs fighting the tumor, and (4) TNFa and IL-2 would also recruit further virus-loaded TILs in a self-amplifying loop.

## 2. Materials and Methods

### 2.1. Cell Lines and Viruses

Hamster HapT1 pancreatic tumor cell line was kindly provided by Dr Hernandez-Alcoceba (Pamplona, Spain) and maintained in RPMI supplemented with 10% fetal bovine serum (FBS), 1% L-Glutamine, and 1% Pen/Strep solution (Sigma-Aldrich, St. Louis, MO, USA) at 37 °C and 5% CO_2_. Ad5/3-Luc1 [17], Ad5/3-E2F-d24-hTNFa, Ad5/3-E2F-d24-, Ad5/3-E2F-d24-hTNFa-IRES- (also known as TILT-123) have been previously described [18].

### 2.2. Generation of Human Ovarian Cancer and Hamster Pancreatic TILs

Human ovarian cancer TILs were generated using an adaptation of the “young TILs” protocol [18,19]. In brief, ovarian cancer tumor samples were diced into small fragments and put into a 6-well plate with TIL medium (TM) containing RPMI 1640, supplemented with 20% FBS, 1% L-glutamine, 1% Pen/strep, 15 mM HEPES, 1 mM Na-pyruvate, 50 µm b-Mercaptoethanol (Sigma-Aldrich) and 3000 IU/mL recombinant human IL-2 (Peprotech, Stockholm, Sweden). On day 7, TILs were harvested and frozen up to −140 °C. When thawed, TILs were recovered in TM and subsequently activated with irradiated allogeneic peripheral blood mononuclear cells (PBMC) (40Gy; ratio 1:200 TIL: PBMCs), anti-CD3 antibody (30 ng/mL; ebioscience™, Thermo Fisher Scientific, Waltham, MA, USA) and rapid expansion (RM) medium containing RPMI supplemented with 20% FBS, 1% L-glut, 1% Pen/strep and 3000 IU/mL recombinant human IL-2. On day 5, half of the medium was replaced by a 1:1 mixture of TM and RM and, on day 7, 75% of the medium was replaced by RM medium. After that, the number of TILs was adjusted to 5–10 × 10^6^ TILs/cm^2^ every 3–4 days until day 14.

Hamster TILs were generated using a protocol previously developed by our laboratory [20]. In brief, fully established HapT1 tumors were collected from Syrian hamster, excised in small fragments and cultured in TM for 10 days. Concanavalin A (Sigma-Aldrich) was added to the culture media at day 5.

### 2.3. Quantification of Oncolytic Adenoviruses Bound to Human and Hamster TILs and Tumors

Frozen human ovarian cancer TILs were thawed and incubated with Ad5/3-Luc1, Ad5/3-E2F-d24-hIL2, Ad5/3-E2F-d24-hTNFa and Ad5/3-E2F-d24-hTNFa-hIL2 (TILT-123) at 10 VP/cell in 1 mL of PBS at 37 °C with continuous shaking. After 30 min, the samples were centrifuged at 2000× *g* for 10 min and cell pellet and supernatant were collected. Alternatively, 1 × 10^6^ hamster TILs were thawed and incubated with TILT-123 at 100 VP/cell at 37 °C in 250 µL of RPMI. After 30 min, the samples were centrifuged at 660× *g* for 1 min and subsequently washed with 12 mL of PBS. The cell pellets were washed up to five times with PBS. In washing experiments with human TILs, a sample from the cell pellet and supernatant was collected each time. In washing experiments with hamster TILs, the samples were collected from individual tubes assigned a number of washes (up to 5). Each washing experiment had one sample per wash. QIamp DNA kits (Qiagen, Germany) were used to extract DNA from collected samples of cell suspensions or snap-frozen tumor tissue in order to quantify the viral genomes through quantitative PCR (qPCR) [21]. In samples originating from in vivo experiments, the number of viral copies was normalized against the housekeeping gene expression, either mouse β-actin or hamster GAPDH. qPCR was performed in duplicates or triplicates.

### 2.4. Incubation of Oncolytic Virus with Hamster TILs

For animal experiments, hamster TILs were washed by centrifugation and seeded in a 24-well plate containing 1 mL of RPMI supplemented with 1% Pen/Strep and 1% L-Glutamine, on the same day. TILT-123 (ratio 1:100 or 1:500 TIL:VP) was added to the cell suspension and incubated at 37 °C and 5% CO_2_ for 30 min with rocking. Without washing, hamster TILs incubated with TILT-123 were injected in mice. In one instance, hamster TILs were incubated with Qtracker™ 525 Cell Labeling Kit (Thermo Fisher Scientific) according to manufacturer’s recommendations before incubation with virus.

### 2.5. Animal Studies

Assessment on the capacity of TILs loaded with TILT-123 to deliver virus was performed in hamsters and in mice. Immunocompetent Syrian Hamsters (HsdHan:AURA) were implanted with 4 × 10^6^ HapT1 cells in both left and right lower back flanks. After 7 days, hamsters were randomized in four groups of five individuals. On day 0, hamsters received i.t. injections of PBS or 1 × 10^8^ VP/50 μL of TILT-123 only and intraperitoneal (i.p.) injections of 8 × 10^6^ hamster TILs loaded with TILT-123 with 4 × 10^9^ VPs (ratio 1:500 TIL:TILT-123) or 4 × 10^9^ VP/100 μL of TILT-123 alone. On the same day, another set of hamsters received i.t. injections of PBS or 1 × 10^8^ VPs and i.p. injections of 8 × 10^6^ hamster TILs loaded with TILT-123 with 0.8 × 10^9^ VPs (ratio 1:100 TIL:TILT-123) or 0.8 × 10^9^ VPs of TILT-123. The set of hamsters receiving TILs incubated with virus at a ratio of 1:500 and respective controls were euthanized 3 days after beginning of treatments. Hamsters receiving TILs with virus at a ratio of 1:100 and respective controls were euthanized 5 days after the beginning of treatments. 

Immunocompromised NOD.Cg-*Prkdc*^scid^-*Il2rg*^tm1Sug^/JicTac (Taconic, Silkeborg, Denmark) mice were implanted with 4 × 10^6^ HapT1 cells in both left and right lower back flanks. After 6 days of tumor engraftment, mice were randomized in four groups of 3–4 individuals. On day 0, mice received i.t. injections of PBS or 1 × 10^8^ VPs/50 μL and i.v. injections of 5 × 10^6^ hamster TILs loaded with TILT-123 with 2.5 × 10^9^ VPs (ratio 1:500 TIL:TILT-123) or 2.5 × 10^9^ VP/100 μL of TILT-123. Following 4 days after treatment, mice were euthanized, and their tumors were collected and snap-frozen for detection of adenovirus genomes.

To evaluate the antitumor efficacy of TILs loaded with TILT-123 in non-injected tumors, we implanted immunocompromised NOD.Cg-*Prkdc*^scid^-*Il2rg*^tm1Sug^/JicTac (Taconic) with 4 × 10^6^ HapT1 cells in both left and right lower flanks. After 4 days growing, mice were randomized into six groups of 5–6 individuals. On days 0 and 7, left tumors received i.t. injections of PBS or TILT-123 (1 × 10^9^ VP/50 μL) and mice received i.v. injections of 5 × 10^6^ hamster TILs loaded with TILT-123 with 2.5 × 10^9^ VPs (ratio 1:500 TIL:TILT-123), 5 × 10^6^ hamster TILs, 2.5 × 10^9^ VPs of TILT-123, or PBS. Tumor growth was recorded over 11 days. Animals were euthanized whenever tumor size limit was reached or it was ulcerated (clear signs of recent bleeding or structural depressions). The volume of tumors was calculated as 0.52 × length × width^2^. Normalized tumor volume was obtained by normalizing day 8, day 10 and day 11 tumor volumes to the PBS tumor volumes in each respective day.

Each animal experiment was performed once. All the animal protocols were approved by the Provincial Government of Southern Finland and the experimental animal committee of the University of Helsinki.

### 2.6. Tumor Sample Processing and Flow Cytometric Analysis 

Tumor samples from animals were collected, processed through mechanical grinding, and stored at −80 °C until further flow cytometry analysis. Anti-Rat CD8b PE (cat number 12-0080-82, clone eBio341, ebioscience™, Thermo Fisher Scientific) and anti-mouse CD4 APC (cat number 17-0041-82, clone GK1.5, ebioscience™, Thermo Fisher Scientific) were used to stain tumor samples. Stained samples were analyzed on the BD Accuri C6 flow cytometer using the CSampler software (Version 1.0264.15, BD Biosciences, San Jose, CA, USA).

### 2.7. Statistics

GraphPad Prism 8 (graph Pad Prism Software Inc., San Diego, CA, USA) was used to generate graphs for data representation and to assess differences between experimental groups with regular one-way ANOVA with Tuckey’s multiple comparison test or Kruskal–Wallis with Dunn’s multiple comparisons and unpaired Student’s *t*-test with Welch’s correction. In the mouse efficacy experiment, statistical differences between therapeutic groups were assessed on log-transformed absolute tumor volumes or PBS group-normalized tumor volumes using linear mixed models in SPSS version 26 (IBM, North Castle, NY, USA)

## 3. Results

### 3.1. Ad5/3 Oncolytic Adenoviruses Bind to Clinically Relevant Human and Hamster TILs

**Figure 1:**
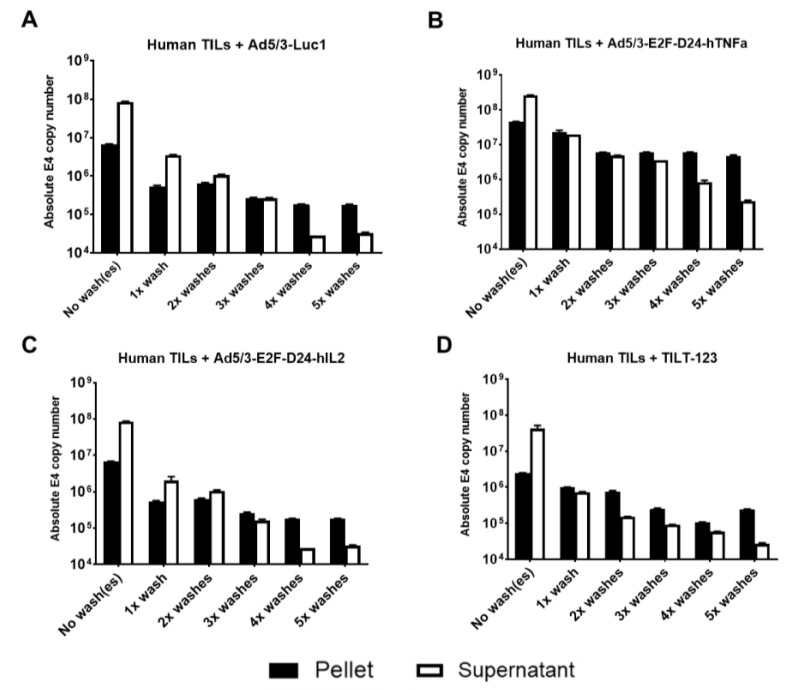
Binding of Ad5/3 oncolytic adenoviruses to clinically relevant human ovarian cancer TILs. Human ovarian cancer TILs were washed, incubated for 30 min with (**A**) Ad5/3-Luc1, (**B**) Ad5/3-E2F-D24-hTNFa, (**C**) Ad5/3-E2F-D24-hIL2 and (**D**) TILT-123, and washed up to five times by centrifugation. DNA was extracted from pellets and supernatants after every wash and analyzed for the presence of virus DNA copies (E4). Data is presented as mean + SEM.

Given the ability of Ad5/3 oncolytic adenovirus to bind lymphocytes [13], we hypothesized that this interaction would be similar for clinically relevant cell subtypes, such as TILs. To address this question, we performed washing experiments with chimeric Ad5/3 oncolytic adenoviruses, including Ad5/3-Luc1, Ad5/3-E2F-d24-hIL-2, Ad5/3-E2F-d24-hTNFa and TILT-123, and human ovarian cancer TILs. Washing removes virus from cells into the supernatant, and any virus that reappears after washing into fresh supernatant represents virus that detached from TILs. The washing could also represent (to some extent) the dynamic mechanical forces exerted by the blood stream when TILs are travelling in vivo. The fact that virus reappeared in supernatant even after five washes demonstrated the dynamic nature of the binding; virus could spontaneously detach from TILs (Figure 1). On the other hand, because virus remained in the pellet event after five washes shows that the virus is not easily completely detachable from the cells. Interestingly, the binding avidity of the different viruses is seemingly different despite having the same capsid. This may be due to the technical aspects of the assay (each panel represents an independent run) or due to the unit of measurement used in the assay (VP). While we used the same number of VPs per tumor cell in our assays, it is conceivable that the viruses had different measurements of plaque-forming units. This means that the actual number of virus particles that contained actual viral DNA may differ within each virus, thus accounting for the differences seen.

### 3.2. Hamster TILs Binding TILT-123 Deliver TILT-123 to Subcutaneous Tumors Following Intraperitoneal Injection

Considering that TILs have shown the ability to reach tumors after systemic administration and to induce an antitumor response [22,23], we hypothesized if TIL functionality could be expanded to act as cell carriers of Ad5/3 viruses for tumor delivery. 

**Figure 2:**
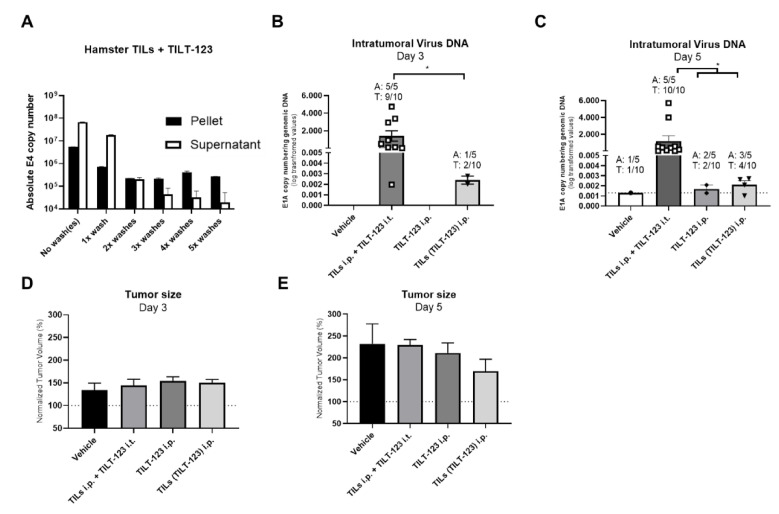
Binding of TILT-123 to hamster TILs, virus copy numbers in HapT1 tumors and antitumor efficacy in hamster tumors. (**A**) After expansion, hamster TILs were washed, incubated for 30 min with TILT-123 and washed up to five times. DNA was extracted from pellets and supernatants after every wash and analyzed for the presence of virus DNA copies (E4); (**B**,**C**) hamsters bearing bilateral HapT1 tumors were treated with i.t. injections of TILT-123 or PBS and i.p. injected with TILs loaded with TILT-123 [TILs(TILT-123)], TILT-123 or RPMI. DNA was extracted from (**B**) day 3 or (**C**) day 5 snap-frozen tumors and analyzed for the presence of virus DNA copies (E1A). The virus copy numbers were normalized to the amount of genomic DNA in the samples (GAPDH expression); hamsters bearing bilateral HapT1 tumors were treated with i.t. injections of TILT-123 or PBS and i.p. injected with TILs loaded with TILT-123 [TILs(TILT-123)], TILT-123 or RPMI. Tumors were measured with an electronic caliper on days 3 (**D**) and 5 (**E**). Normalized tumor volume data was obtained by normalizing the daily tumor volume with day 0 tumor volumes. Statistically significant differences in (**C**) were assessed using Kruskal Wallis test with Dunn’s multiple comparison test while in (**B**) were assessed using unpaired Student’s *t* test with Welsh’s correction. No statistically significant differences were found among the groups by using one-way ANOVA with Tukey’s multiple comparison test in (**D**,**E**). Only statistically significant differences are shown. Data is presented as mean + SEM; A—animal, T—tumor. *—*p* < 0.05.

The first step to investigate this in vivo was to confirm that TILT-123 can bind to hamster TILs similarly to human TILs. Using the previous experimental set up, we confirmed that consecutive washings do not ablate the presence of virus in the hamster TILs (Figure 2A). The first two washes significantly reduced the number of virus in the hamster TIL pellets, while subsequent washes induced no meaningful decrease of virus copies in hamster TIL pellets (Figure 2A).

Next, we evaluated virus tumor delivery in vivo after preincubation with TILs. We then injected TILs incubated with TILT-123 (TILs(TILT-123) i.p.) into hamsters, followed by determination of virus DNA in subcutaneous hamster tumors. Control groups included i.p. and i.t. injections of TILT-123 (with TILs given i.p. in the latter group). As expected, tumors injected i.t. had higher virus copy number (per genomic DNA) at days 3 and 5 than the other groups (average ≈ 1.4 and 1.2 of E1A copies/ng of genomic DNA, respectively; Figure 2B,C). Of note, virus DNA was present in tumors of animals receiving TILs loaded with TILT-123, a phenomenon seen in 20% of the animals at day 3 and in 60% of the animals at day 5 (average 0.0024 and 0.0021 of E1A copies/ng of genomic DNA, respectively; Figure 2B,C). These numbers were higher than those resulting from i.p. injection of virus alone. Thus, i.p. injected TILs were able to deliver TILT-123 to distant tumors. The presence of virus DNA in vehicle tumors, similar in crossing point (CP) values to TILT-123 i.p. and TILs(TILT-123) i.p. groups, may relate to technical limitations of the tumor sampling, including the minimal risk of cross-contamination.

Interestingly, some signs of antitumor activity could already be seen on day 5 in the group where TILT-123 was delivered with TILs, relative to animals administered with vehicle (Figure 2E). Of note, no tumor volume bias was found between the groups after randomization (Supplementary Figure S1).

### 3.3. TILT-123-Loaded Hamster TILs Deliver TILT-123 into Tumors through the Intravenous Route and Induce the Infiltration of Further TILs

Considering that in humans the i.v. route is preferred for systemic delivery, we looked for a second animal model in which this route was more feasible (hamsters lack a tail vein), such as mice. However, mouse cells are biologically non-permissive for human adenovirus replication. To circumvent this issue, we utilized immunocompromised NOG mice engrafted subcutaneously with hamster HapT1 tumors (which are permissive for adenovirus replication), infused with hamster HapT1 TILs for biodistribution studies. Mice were treated i.v. with TILs loaded with TILT-123. Control groups received TILT-123 i.v. (TILT-123 i.v.) or i.t. simultaneously with i.v. injection of TILs (TILT-123 i.t. + TILs i.v.). I.t. injection of TILT-123 together with i.v. administration of TILs is the current clinical setting in a Phase 1 trial in melanoma patients (NCT04217473).

**Figure 3:**
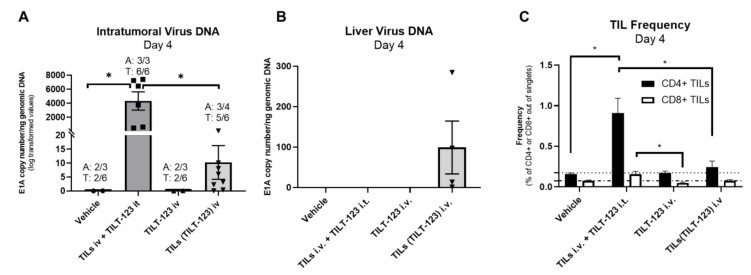
Biodistribution of virus following treatment. Immunocompromised NOG mice bearing bilateral HapT1 tumors were treated with i.t. injections of TILT-123 or PBS and i.v. injected with hamster TILs loaded with TILT-123 [TILs(TILT-123)], TILT-123, or PBS. DNA was extracted from day 4 snap-frozen (**A**) tumors and (**B**) livers and analyzed for the presence of virus DNA copies (E1A). The virus copy numbers were normalized to the amount of genomic DNA in the samples (GAPDH expression). (**C**) Tumors were collected, processed, and analyzed by flow cytometry for CD4 and CD8 positive cells. Dashed lines indicate the highest average background in control groups for CD8+ and CD4+ cells. Statistically significant differences were found by performing Kruskal Wallis test with Dunn’s multiple comparison test. Only statistically significant differences are shown. Data is presented as mean + SEM; A—animal, T—tumor. *—*p* < 0.05.

Again, as expected, the current standard administration method (TILs i.v. + TILT-123 i.t.) resulted in high levels of *E1A* copies in tumors from 100% of the animals (average = 0.9 of E1A copies/ng of genomic DNA; 3/3 mice, 6/6 tumors; Figure 3A). This increase was statistically significant compared to vehicle and TILs(TILT-123) i.v. treated groups (both *p* < 0.05; Figure 3A).

Importantly, administration of TILs loaded with virus [TILs(TILT-123) i.v.] led to detectable virus copy numbers in most tumors (average 11.7 of E1A copies/ng of genomic DNA; 3/4 mice, 5/8 tumors; Figure 3A). Mice in the TILs(TILT-123) i.v. group accumulated the highest copy numbers of virus in the liver (average 99 of E1A copies/ng of genomic DNA, Figure 3B). This suggested that some of the TILs loaded with virus got stuck in liver sinusoids. 

To assess the frequency of transferred TILs, we used CD4 and CD8b antibodies cross-reactive known to cross-react with the corresponding markers in hamsters. This is the preferred approach considering that anti-hamster CD3 antibodies are not commercially available. We noted some background expression of CD4 and CD8b in test groups not receiving TILs, possibly due to cross-reaction of antibodies to other endogenous cell types (Figure 3C). This mouse model lacks mature T, B and NK cells, however, it possesses dysfunctional DCs, which can also express CD4. It is unclear from the literature that anti-rat CD8 cross-reacts with murine immune cells. Nevertheless, we saw a significant increase in the proportion of CD4+ (average ≈ 0.91%) and CD8+ cells (average ≈ 0.18%) in tumors from animals treated with TILs i.v. + TILT-123 i.t. compared with the PBS and TILs(TILT-123) (both *p* < 0.05; for CD4+ cells) and TILT-123 i.v. (*p* < 0.05; for CD8+ cells). Thus, TILT-123 i.t. was able to recruit TIL-like populations into tumors. In contrast, tumors from animals receiving hamster TILs loaded with TILT-123 showed a much smaller increase in the proportion of the levels of CD4+ (average ≈ 0.25%) and CD8+ cells (average ≈ 0.08%), is compatible with virus-loaded TILs becoming stuck in the liver (Figure 3C).

### 3.4. Anti-Tumor Efficacy of TILs Loaded with TILT-123 

Next, we sought to investigate if the i.v. administration of TILs(TILT-123) i.v. could result in antitumor efficacy. The setup used was similar to the aforementioned biodistribution experiment, except that in the i.t. control groups only the left tumor was treated with TILT-123 or PBS. Treatments were performed once a week (days 0 and 7).

Over a course of 11 days, statistically significant tumor size differences were seen between treatment groups (Supplementary Figure S2). As expected, tumors locally treated with TILT-123 presented significant tumor regression compared with control (TILT-123 i.t. vs. all groups except TILs i.v. + TILT-123 i.t., at least *p* < 0.05), however, infusion of TILs in animals receiving TILT-123 i.t. did not improve the therapeutic outcome of these tumors relative to the monotherapy of TILT-123 (Supplementary Figure S2A). In non-injected tumors, most therapies were capable of significantly decreasing tumor volume over time compared with the PBS treated group (Supplementary Figure S2B).

**Figure 4:**
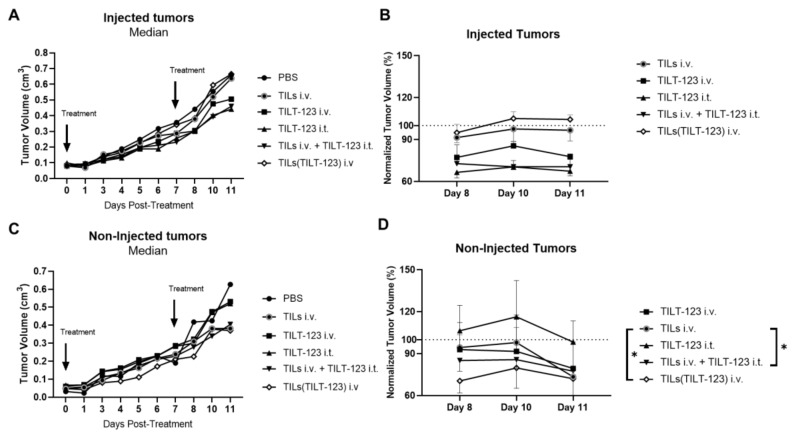
Antitumor efficacy following treatment with hamster TILs loaded with TILT-123. Immunocompromised NOG mice bearing bilateral HapT1 tumors were treated with i.t. injections of TILT-123 or PBS and i.v. injected with TILs loaded with TILT-123 TILs(TILT-123) 1:500 TILs:VP, TILT-123, or PBS once a week. (**A**) Growth of injected tumors over 11 days. Data is presented as median absolute tumor volume (cm^3^); (**B**) growth of injected tumors normalized to PBS control group’s tumor volumes from days 8–11, which means that PBS tumor volumes are set as 100%. (**C**) Growth of non-injected tumors over 11 days. Data is presented as median absolute tumor volume (cm^3^); (**D**) growth of injected tumors normalized to PBS control group’s tumor volumes from days 8–11. Statistically significant differences in (**B**,**D**) were found by performing linear mixed model test in log-transformed PBS-normalized tumor volumes. Only statistically significant differences are shown. Data in (**B**,**D**) is presented as mean + SEM. *—*p* < 0.05.

When we focused on the injected tumors, only trends in median absolute tumor volumes were observed between the therapeutic groups (Figure 4A). When we analyzed the last 3 days of the experiment (after animals completed two rounds of treatment), a trend for anti-tumor activity (not statistically significant) was seen in the positive control groups: TILs i.v. + TILT-123 i.t. and TILT-123 i.t (Figure 4B). Interestingly, the non-injected median absolute tumor volumes from mice treated with TILs loaded with TILT-123 [TILs(TILT-123)] appeared the smallest, although not statistically significant (Figure 4C). During the last 3 days of the experiment, mice treated with TILs(TILT-123) i.v. presented low stable tumor volumes well below PBS (set as 100%) and apparent faster efficacy compared to the other therapeutic groups (Figure 4D). Here, both TILs(TILT-123) i.v. and TILs i.v. + TILT-123 i.t. behaved similarly demonstrating a similar statistical significant difference compared with TILs i.v. group (both *p* < 0.05; Figure 4D).

## 4. Discussion

The combination of cellular therapies with oncolytic viruses can produce attractive benefits for cancer therapy. For example, the use of mesenchymal stem cells (MSCs) was previously shown to improve the delivery oncolytic adenoviruses into tumors in preclinical animal models [24] and humans [25]. Much like MSCs, T-cells can equally deliver oncolytic viruses into tumors with the therapeutic added value of providing tumor-specific cytotoxicity, in the form of antigen-specific T-cells, CAR-T cells or TILs. Other laboratories have indeed loaded viruses, such as vesicular stomatitis virus (VSV), herpes simplex virus (HSV), and vaccinia virus (VV), inside antigen-specific T cells or CAR-T cells for protection from neutralizing antibodies [26,27,28]. Our laboratory, however, focused on using TILs as cell carriers because: (1) TIL therapy has demonstrated impressive efficacy results in humans [29], (2) chimeric 5/3 adenoviruses can bind to lymphocytes [13], (3) TNFa and IL-2 coding oncolytic adenoviruses previously demonstrated excellent synergy with TIL therapy [17], (4) preclinical models have shown that TILs have the ability to home preferentially to tumor sites in contrast with regular T cells [23]. Loading TILs with TILT-123 would potentially lead to enhanced systemic delivery with the synergistic effects of TIL induced antitumor efficacy. In addition, this combined single i.v. injection method might reduce time of infusion in the clinic, as well as hospitalization time, because guided i.t. injections of viruses would not be required. I.t. injection may be challenging at smaller hospitals as they require more logistics than standard i.v. delivery. Thus, TIL mediated delivery of virus would solve a major practical obstacle. Moreover, this approach could protect the virus from neutralizing antibodies [13]. Our results support our hypothesis that TILs can be used as a delivery vehicle of adenoviruses. This was demonstrated, with varying degree of success, in two different animal models, the immunocompetent Syrian hamster and immunocompromised NOG mice, utilizing an intraperitoneal and intravenous mode of administration, respectively.

Interestingly, virus loading may work differently for 5/3 chimeric adenoviruses as opposed to other viruses. While VSV, VV and HSV appear to enter T-cells upon loading, adenoviruses are likely to only bind the surface of T-cells. Previous work from our laboratory showed that PBMCs are resistant to recombinant serotype 5 adenovirus infection [30]. Desmoglein-2, the proposed primary entry receptor from 5/3 chimeric oncolytic adenoviruses, is also minimally expressed on T-cells [31], presumably preventing adenovirus transduction. Transduction of T-cells can be achieved by inserting an RGD-fiber in the HI-loop of the fiber [32]. These viruses have been shown to transduce several hematological cells, such as DCs and T cells, for immunomodulative gene delivery. Instead, we have shown that 5/3 chimeric adenovirus binds to the membrane of lymphocyte, from where it can easily be released into tumors [13]. This might be advantageous over virus types which enter T-cells, as it is not clear how such virus gets out—oncolytic viruses should not lyse non-cancer cells.

Alternative serotype 3 attachment receptors such as CD86 might explain binding to TILs in absence of desmoglein-2, considering that IL-2-mediated activation of human T-cells upregulates CD86 [33]. In this context, the use of IL-2 during TIL culture could represent an advantage for enhanced carrying of TILT-123.

Overall, our approach resulted in some degree of possible tumor reduction, which may derive directly from the combination of antitumor activity and changes in the immune cell composition. Indeed, we have previously found that the combination of TILT-123 and TILs in tumor-bearing immunocompetent Syrian hamster modulates the composition of immune cells [18] and induces the infiltrations of TILs [34,35] in the tumor microenvironment of this animal model. The increased infiltration of TILs (and possibly other subsets) coupled with the reduction of tumor volumes (even at early time points), is indicative of increased antitumor activity in this model. Considering the previous data from our laboratory, we expected a similar behavioral pattern our experiments from Figure 2. Other cell types other than TILs, may have also influenced the tumor volumes at early time points, particularly myeloid cells. In humans, myeloid cells are a large component of the tumor microenvironment of pancreatic cancer [36]. Assuming that hamster HapT1 tumors have similar composition, any impact on such population utilizing TILs and TILT-123 could ultimately reflect on tumor volume.

Nevertheless, the preclinical models used herein would benefit from further optimization. The lack of good models is a common issue in the field of cell carriers and oncolytic adenoviruses [37]. One option could be using patient-derived xenografts (PDX), but they are difficult to generate in NOG mice and this approach would also require human TILs, which are not always readily available for research. On the other hand, human serotype 5 adenovirus is not oncolytic in mouse cancer cells [38]. Therefore, we used hamster HapT1 TILs which have proven antitumor capabilities in HapT1 tumors [20]. Yet, hamsters do not possess a tail vein which prevents practical i.v. injection of cells, leading us to use i.p. administration. Another caveat of the model relates to the lack of commercially available reagents for accurate in-depth analysis of immune the immune system. Some level of tumor microenvironment characterization can often be achieved by genome and transcriptome-based technologies (including granzyme b/IFNg), flow cytometry and simple tumor cell killing assays [17,39] but these are not cell specific.

To get a picture of the effects of i.v. injections on delivery, we used immunocompromised NOG mice bearing bilateral subcutaneous HapT1 xenograft tumors, with hamster TILs given i.v. This model indicated that the use of TILs as carriers might improve the delivery of virus into the tumors over systemic injection of virus alone. In a distinct experiment, we saw some degree of improvement of control of growth of non-injected distant tumors. Nevertheless, it is clear that this model does not fully recapitulate the efficacy of either oncolytic adenoviruses or TIL therapy seen in immunocompetent hamsters [13,17,34,40].

Reasons behind the differing results in preclinical models using TILs can be many. For example, it has been shown that expanding TILs from tumor tissue creates a mostly activated population of tumor reactive T cells. However, there is a variable degree of irrelevant tumor-specific T cells/relevant tumor-specific T cells in the expanded TIL pool of cells. In a study conducted on TILs expanded from a pancreatic cancer, it was shown that more than 50% of CD8^+^ pancreatic TILs were positive for IFN-γ expression, while greater than 70% of CD8^+^ TILs were capable of producing either CD107a or IFN-γ when stimulated. Additionally, a significant amount, but not all cells produced IFN-γ when presented with HLA matched pancreatic cells [41]. This indicates that there is a population of TILs that are not significantly reactive and might benefit from activation by mature antigen-presenting cells (APCs), such as dendritic cells (DC). Since dendritic cells are not functional in immunocompromised mice, this might have affected the results in this study leading to a modest effect of TIL therapy measured in our model. This notion is further supported by reports correlating a successful clinical outcome of TIL therapy and the ability of the transferred T cells to undergo post-infusion priming and expansion. Additionally, antigen presentation and activation of DCs in the tumor-draining lymph node played a role. Therefore, some antitumoral activity in the immunocompetent model could be seen, while the result was modest in immunodeficient models [42].

Furthermore, when tumor blood vessels of PDX model mice have been investigated, a varying degree of human and mouse specific epithelial cells in the vessels was shown, as well as varying and atypical amounts of growth factors and cytokines needed for vascular growth and stabilization [43]. Due to the plausible mismatch between hamster and mice molecules, the vascularization of the tumor might be poor, and adhesion and homing molecule expression might be different. Therefore, human TILs might struggle to enter the tumor from the bloodstream in PDX models.

It is of potential importance that the biological dynamics the approach described herein could be leveraged for delivering adenovirus to specific anatomical regions where cancer may be present. The fact that our experiments showed that increased copy numbers of virus were seen in the liver from animals treated with TILs loaded with TILT-123 may hint that the approach can be useful to deliver virus in the context of primary or metastatic cancer in the liver. Similarly, the approach can also be useful to deliver virus into lung tumors. Preclinical data shows that adoptively transferred T-cells tend to persist in the lungs in the first hours of post-infusion [44].

In summary, we showed that TILs can be used for delivery of clinically relevant oncolytic adenoviruses, such as TILT-123, into tumors. However, we were not able to identify a preclinical model which would capture all the relevant aspects of the human situation. While some antitumor activity was seen in non-injected tumors, a more emphatic preclinical demonstration of efficacy might be useful for human translation. Nevertheless, the approach remains promising for further optimization of use of TILT-123, and potentially other oncolytic adenoviruses, in the context of TIL therapy.

## Data Availability

Restrictions apply to the availability of these data.

## Supplementary Materials

The following are available online at https://www.mdpi.com/article/10.3390/cells10050978/s1, Figure S1: Initial Hapt1 tumor volumes, Figure S2: Average tumor volumes of animals treated with TILT-123 and TILs.
