# Supplementary material for: Systemic Delivery of Oncolytic Adenovirus to Tumors Using Tumor-Infiltrating Lymphocytes as Carriers
Source: Cells. 2021 Apr 22;10(5):978. doi: 10.3390/cells10050978 (PMC8143525; doi:10.3390/cells10050978)
Supplement: Supplementary file 1 [file cells-10-00978-s001.zip › cells-1150083-supplementary.pdf]

# Supplementaty Figure 1

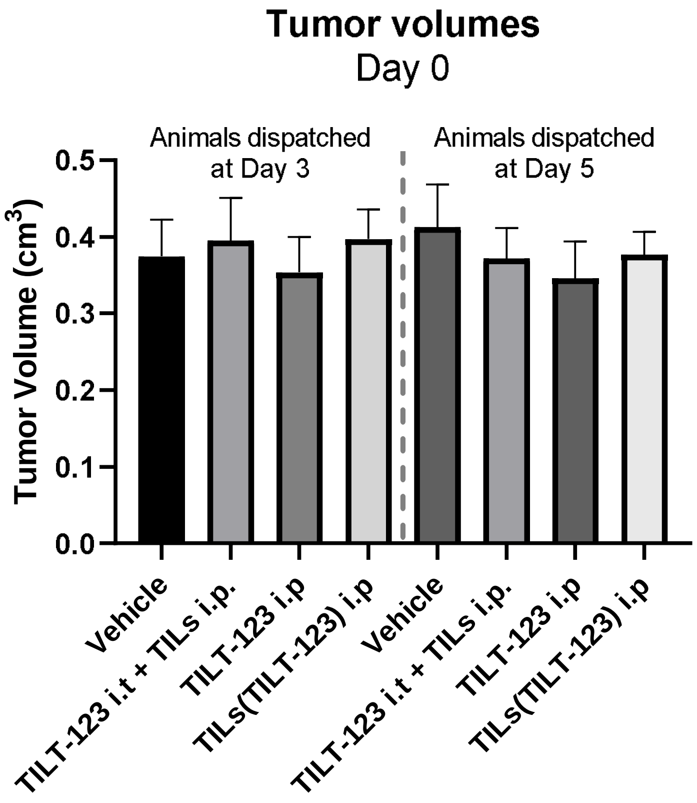


Supplementary Figure 1. Initial Hapt1 tumor volumes. Tumors (2 per animal) from treated animals (n=5-6) were measured with a digital caliper at day 0. No statistically significant differences were found among the groups by using one-way ANOVA with Tukey test.

# Supplementaty Figure 2

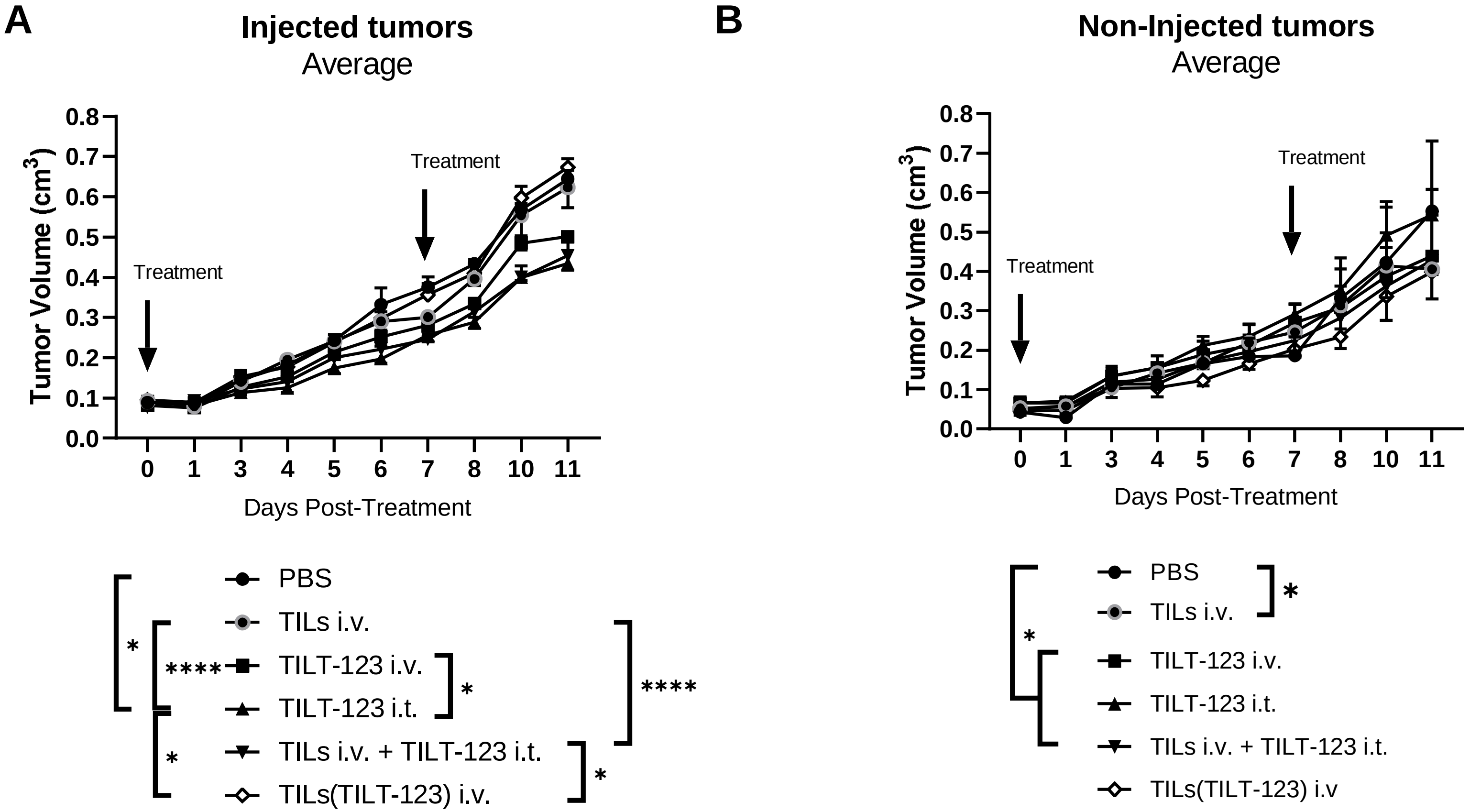


Supplementary Figure 2 – Average tumor volumes of animals treated with TILT-123 and TILs. Immunocompromised NOG mice bearing bilateral HapT1 tumors were treated with i.t. injections (only left tumor) of TILT-123 or PBS and i.v. injected with TILs loaded with TILT-123 TILs(TILT-123) 1:500 TILs:VP, TILT-123, or PBS once a week. (A) Growth of injected tumors over 11 days. (B) Growth of non-injected tumors over 11 days. Statistically significant differences were found by performing linear mixed model test in log-transformed absolute tumor volumes. Only statistically significant differences are shown. Data is presented as mean + SEM. *-p<0.05,**-p<0.01, ****-p<0.0001
